# Supplementary material for: A DNA alteration and methylation co-detection method for clinical purpose
Source: EMBO Mol Med. 2025 Jun 4;17(7):1825–41. doi: 10.1038/s44321-025-00259-7 (PMC12254327; doi:10.1038/s44321-025-00259-7)
Supplement: Supplementary file 3 — Appendix [file 44321_2025_259_MOESM3_ESM.pdf]

Appendix

A DNA alteration and methylation co-detection method for clinical purpose

Jiyan Yu *et al.*

*Corresponding author. Email: shuang.yang@amoydx.com

Table of contents

**Appendix Figure S1**	Assessment of various modified dCTP as protective base in MM-seq	Page 2
**Appendix Figure S2**	Comparison of methylation assessment by EM-seq and MM-seq	Page 3
**Appendix Figure S3**	Comparison of genomic sequence evaluation between WGS and MM-seq	Page 4
**Appendix Figure S4**	Application of MM-seq in real world samples	Page 5
**Appendix Figure S5**	Measurement of methylation-inferred immune-derived DNA by MM-seq	Page 6
**Appendix Figure S6**	Identification of biallelic loss of function in HRR genes using MM-seq	Page 7
**Appendix Table S1**	Primary and converted UMI sequences used in MM-seq	Page 8
**Appendix Table S2**	Gene list in LC10 and LC27 panel	Page 9
**Appendix Table S3**	NSCLC-related methylation markers	Page 10
**Appendix Table S4**	Tumor detection with standard DNA-seq and MM-seq	Page 11
**Appendix Table S5**	Comparison of time cost in mutation and methylation identification between techniques	Page 12

Appendix Figure S1.

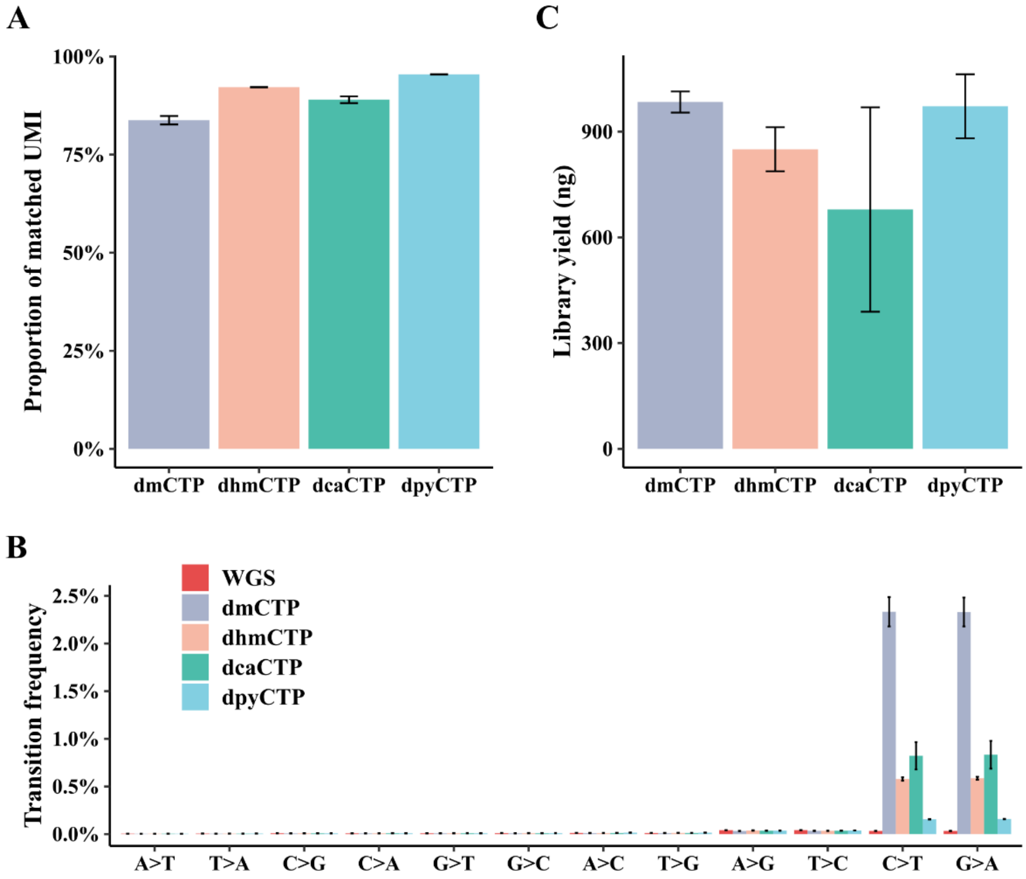

Appendix Figure S1. Assessment of various modified dCTP as protective base in MM-seq. Four types of modified dCTP (dmCTP, dhmCTP, dcaCTP, and dpyCTP) were evaluated in MM-seq to generate co-detection libraries using genomic DNA from the NA12878 cell line, with three replicates for each type. **(A)**, proportions of UMIs perfectly matching the designed sequences in MM-seq libraries generated with different dCTPs. **(B)**, frequencies of cytosine transitions introduced by different types of modified dCTP compared to regular dCTP in WGS. **(C)**, library yields in MM-seq libraries using different dCTPs under identical PCR conditions. Data are presented as mean ± standard deviation in (**A**, **B** and **C**).

Appendix Figure S2.

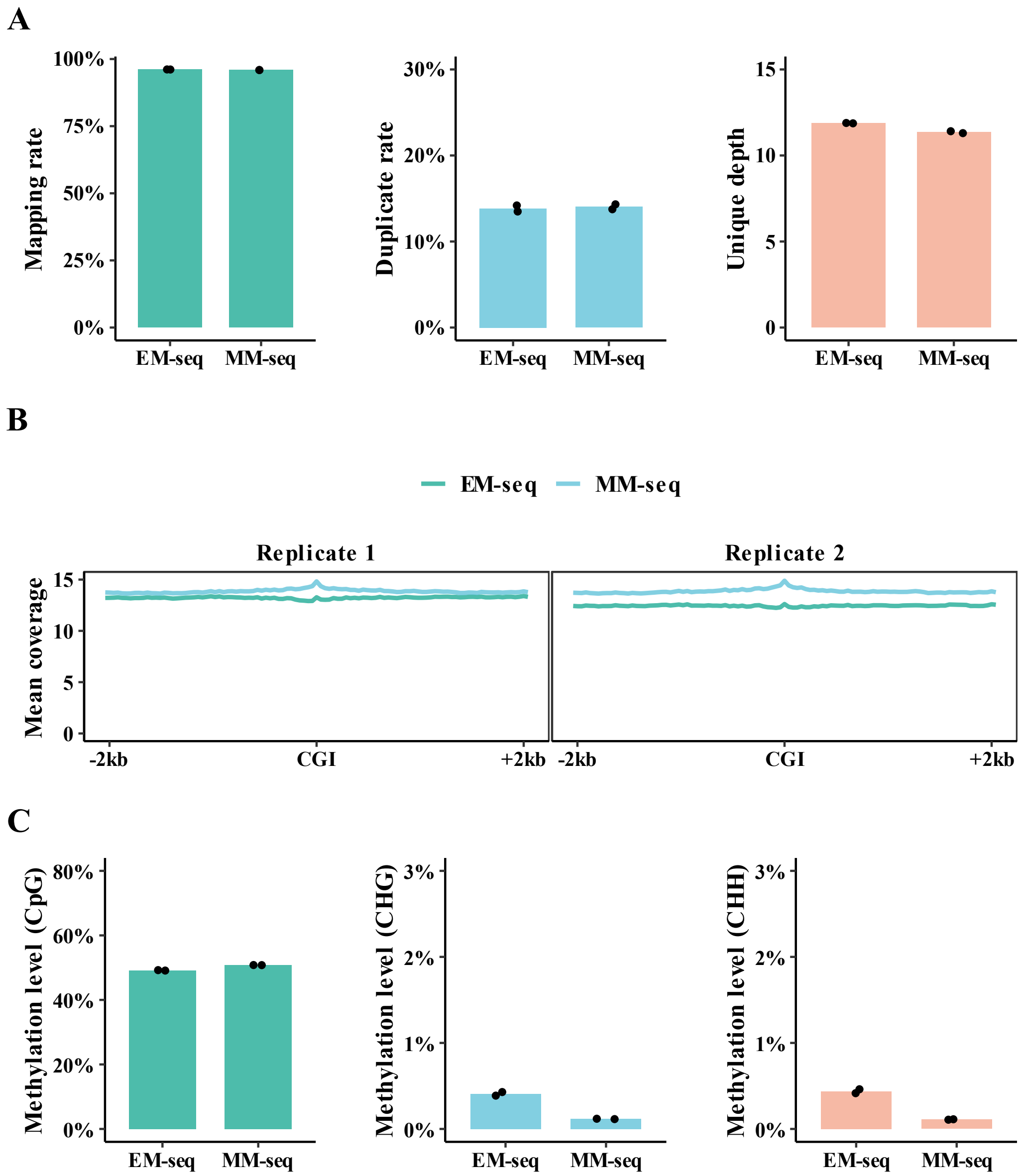

Appendix Figure S2. Comparison of methylation assessment by EM-seq and MM-seq. Methylation analysis was conducted in duplicate using genomic DNA from the NA12878 cell line (n = 2), comparing MM-seq and EM-seq. **(A)**, proportions of reads mapped to the genome, proportions of DNA fragment duplicates, and valid depth after duplicate removal were collected for each method, with mean values from the two replicates plotted. **(B)**, coverage of CpG islands and their flanking regions by EM-seq and MM-seq. The mean unique depth of all CpG islands across the genome, including their 2 kb flanking regions (divided into 50 windows), was plotted. **(C)**, beta values of all cytosines within CpG, CHG, and CHH contexts were calculated and averaged for each method using data from the two replicates.

Appendix Figure S3.

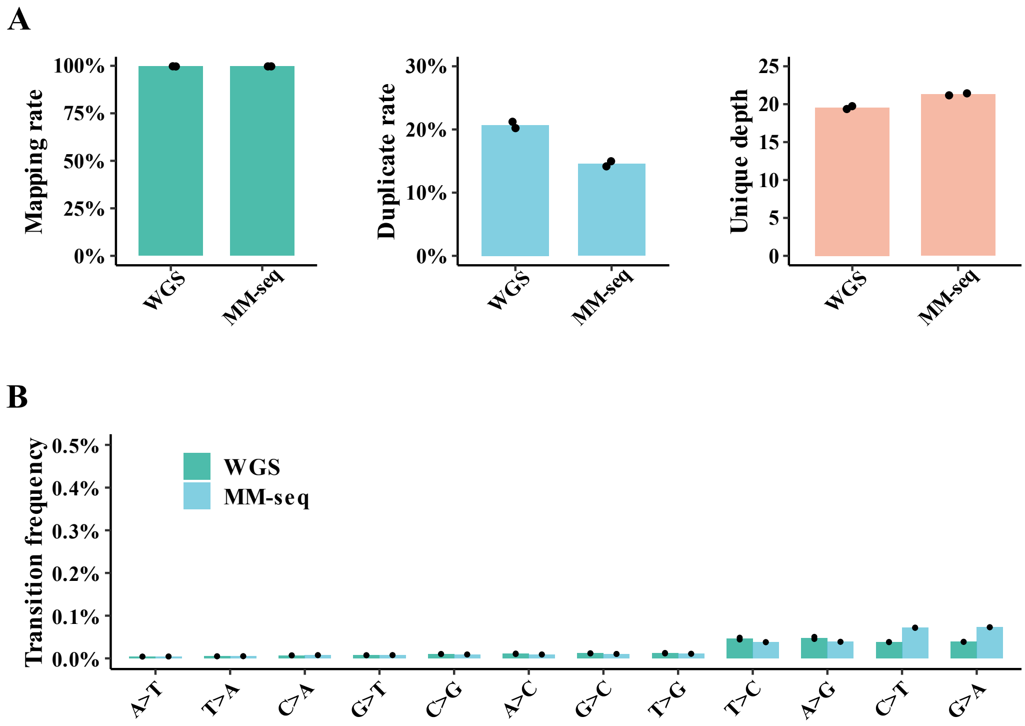

Appendix Figure S3. Comparison of genomic sequence evaluation between WGS and MM-seq. Genomic alteration analysis was conducted in duplicate using genomic DNA from the NA12878 cell line (n = 2), comparing MM-seq and WGS. **(A)**, proportions of reads mapped to the genome, proportions of DNA fragment duplicates, and valid depth after duplicate removal were collected for each method, with mean values from two replicates plotted. **(B)**, comparison of base substitution rates between WGS and MM-seq. The figure shows the mean frequencies of base substitutions observed across the exome in two replicates of NA12878 cell line for each method.

Appendix Figure S4.

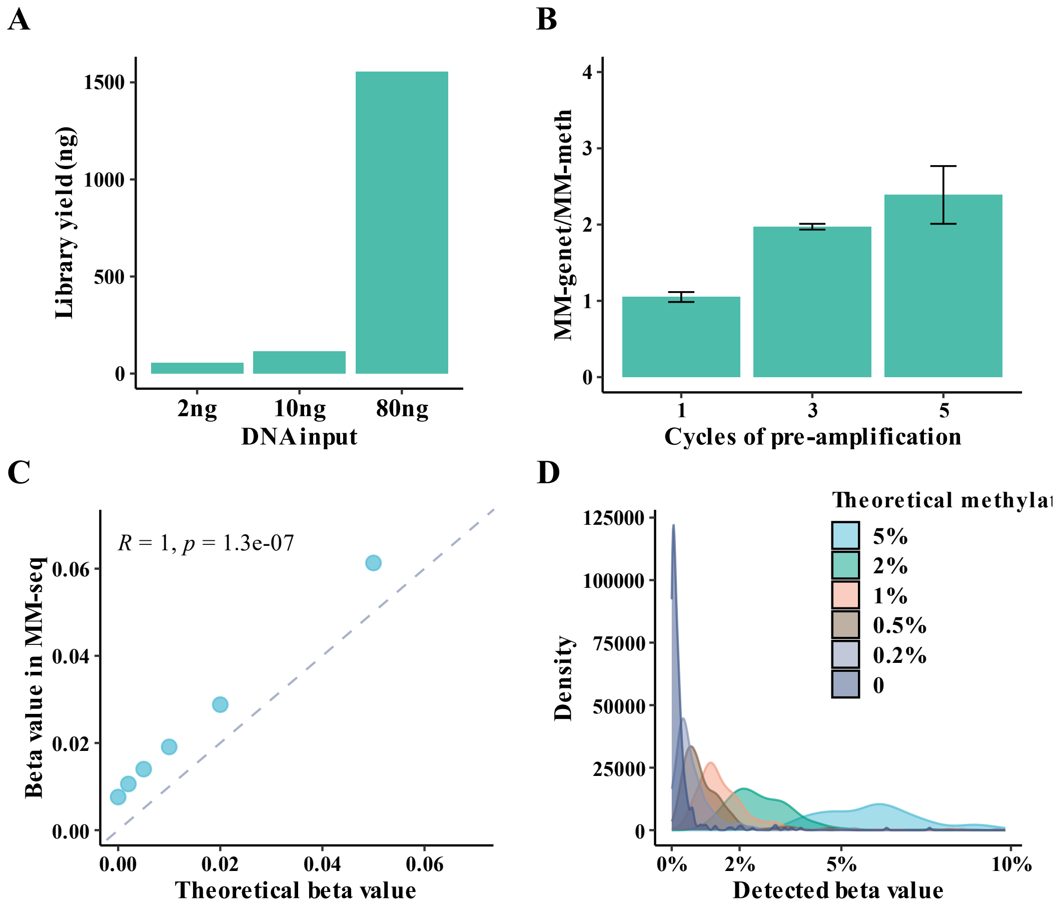

Appendix Figure S4. Application of MM-seq in real world samples. (A), library yields generated by MM-seq using 2 ng and 10 ng cfDNA, as well as 80 ng genomic DNA input, under 7 PCR cycles. **(B),** ratio of reads in MM-genet to MM-meth using different cycles of copy strand synthesis in a mixed cfDNA sample (performed in triplicates), shown as mean ± standard deviation. **(C),** measurement of low methylation levels in cfDNA samples using MM-seq. Six cfDNA samples were spiked with pUC19 DNA at varying methylation levels (5%, 2%, 1%, 0.5%, 0.2%, 0%) and subjected to MM-seq. The correlation between the CpG methylation of pUC19 measured by MM-seq and the theoretical dilution is depicted (t-test for testing the significance of correlation). **(D),** beta value distribution of CpG methylation in the pUC19 genome measured by MM-seq across various dilutions.

Appendix Figure S5.

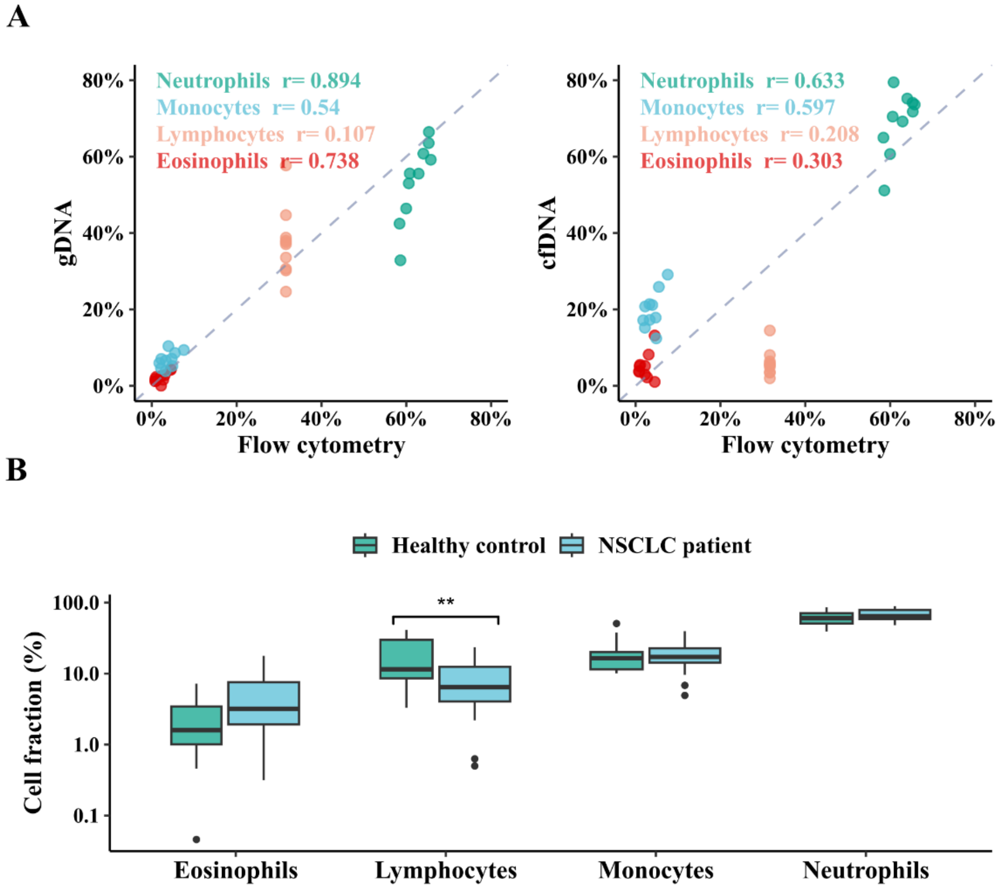

Appendix Figure S5. Measurement of methylation-inferred immune-derived DNA by MM-seq. (A), correlation between immune-derived gDNA and cfDNA inferred from immune cell type-specific methylation and immune cell proportions measured by flow cytometry. **(B)**, comparison of fractions of immune cell subsets calculated with methylation-inferred immune cell-specific cfDNA between NSCLC patients (N = 26) and healthy individuals (N = 13) (Student's t-test, **: p-value = 0.0033). In boxplots, solid black lines show the median, box edges mark the first and third quartiles, whiskers reach the furthest points within 1.5 times the interquartile range, and outliers beyond this range define the minimum and maximum.

Appendix Figure S6.

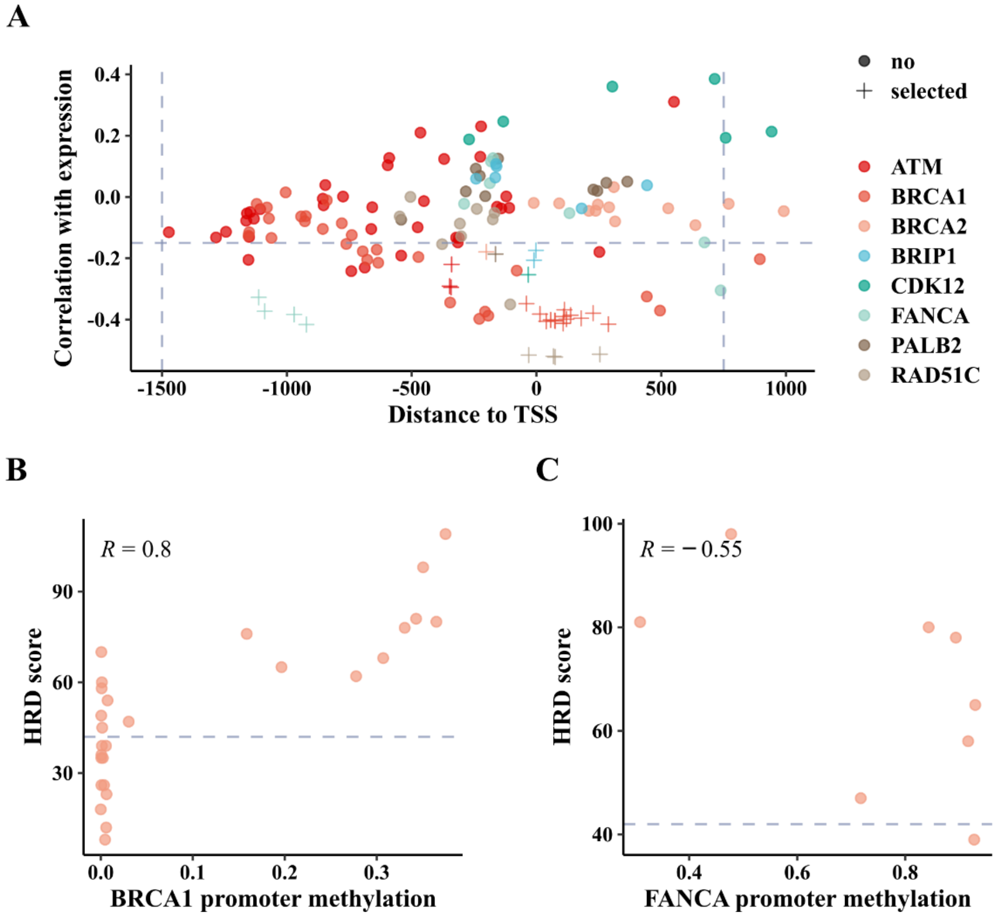

Appendix Figure S6. Identification of biallelic loss of function in HRR genes using MM-seq. (A), screening of expression-correlated CpG sites within the promoter regions of HRR genes. CpG candidates were confined to regions extending 1,000 bp upstream and 750 bp downstream of the transcription start site. CpG sites that were no more than 100 bp apart and had the lowest Pearson's correlation coefficient with gene expression were selected. **(B),** correlation between the promoter methylation level of *BRCA1* and the HRD score. A HRD score of 42 was used as the threshold to classify samples as HRD or HRP. **(C),** correlation between promoter methylation levels of *FANCA* and the HRD score in patients with *FANCA* LOH.

Appendix Table S1.

Primary and converted UMI sequences used in MM-seq

UMI name	UMI sequence	UMI reverse complementary sequence	Converted UMI sequence	Converted UMI reverse complementary sequence
UMI1	GACTCGAT	ATCGAGTC	GATTTGAT	AACTCAAT
UMI2	TTCCACGG	CCGTGGAA	TTTTATGG	TTCCACAA
UMI3	CGCATGAT	ATCATGCG	TGTATGAT	CACATAAT
UMI4	ACGCTACA	TGTAGCGT	ATGTTATA	ACACTACA
UMI5	CGGCTAAT	ATTAGCCG	TGGTTAAT	CAACTAAT
UMI6	GCTATCCT	AGGATAGC	GTTATTTT	ACTATCCT
UMI7	TGGACTCT	AGAGTCCA	TGGATTTT	TAAACTCT
UMI8	ATCCAGAC	GTCTGGAT	ATTTAGAT	ATCCAAAC
UMI9	CTTAGGAC	GTCCTAAG	TTTAGGAT	CTTAAAAC
UMI10	GTGCCTTA	TAAGGCAC	GTGTTTTA	ATACCTTA
UMI11	TCGCTGTT	AACAGCGA	TTGTTGTT	TCACTATT
UMI12	TTCGTTGG	CCAACGAA	TTTGTTGG	TTCATTAA
UMI13	AAGCACTG	CAGTGCTT	AAGTATTG	AAACACTA
UMI14	GTCGACGA	TCGTCGAC	GTTGATGA	ATCAACAA
UMI15	ACCACGAT	ATCGTGGT	ATTATGAT	ACCACAAT
UMI16	GATTACCG	CGGTAATC	GATTATTG	AATTACCA
UMI17	GCACAACT	AGTTGTGC	GTATAATT	ACACAACT
UMI18	GCGTCATT	AATGACGC	GTGTTATT	ACATCATT
UMI19	GACGGACG	CGTCCGTC	GATGGATG	AACAAACA
UMI20	ACTGAGGT	ACCTCAGT	ATTGAGGT	ACTAAAAT
UMI21	TGAACACG	CGTGTTCA	TGAATATG	TAAACACA
UMI22	GTTACGCA	TGCGTAAC	GTTATGTA	ATTACACA
UMI23	AGCGTGTT	AACACGCT	AGTGTGTT	AACATATT
UMI24	GATCGAGT	ACTCGATC	GATTGAGT	AATCAAAT
UMI25	TTGCGATG	CATCGCAA	TTGTGATG	TTACAATA
UMI26	CTGTTGAG	CTCAACAG	TTGTTGAG	CTATTAAA
UMI27	GCTATCTG	CAGATAGC	GTTATTTG	ACTATCTA
UMI28	ACGTTCAG	CTGAACGT	ATGTTTAG	ACATTCAA
UMI29	TTGCAGAC	GTCTGCAA	TTGTAGAT	TTACAAAC
UMI30	CAATGTGG	CCACATTG	TAATGTGG	CAATATAA
UMI31	ACGACTTG	CAAGTCGT	ATGATTTG	ACAACTTA
UMI32	ACTAGCAG	CTGCTAGT	ATTAGTAG	ACTAACAA

Appendix Table S2.

Gene list in LC10 and LC27 panel

Gene name	Prevalence in CHOICE cohort	Prevalence in TCGA cohort	Contained in LC10 panel
EGFR	31.58%	7.80%	Yes
KRAS	8.19%	15.90%	Yes
PIK3CA	5.85%	8.30%	Yes
ERBB2	1.75%	1.60%	Yes
MET	1.17%	2.60%	Yes
RET	1.17%	2.30%	Yes
ALK	0.58%	2.20%	Yes
NRAS	0.58%	0.70%	Yes
ROS1	0.58%	1.50%	Yes
BRAF	0.00%	4.40%	Yes
TP53	47.95%	66.00%	
STK11	9.94%	7.30%	
CDKN2A	9.36%	8.60%	
KEAP1	9.36%	13.40%	
LRP1B	9.36%	0.30%	
RGPD1	8.77%	0.00%	
LRRC37A3	8.19%	0.00%	
AGTPBP1	7.02%	0.10%	
FAT1	5.85%	6.10%	
USP9Y	5.26%	0.00%	
CACNA1E	3.51%	1.00%	
KMT2C	2.92%	0.50%	
ARID1A	1.17%	0.40%	
CBX6	1.17%	0.00%	
ACACB	0.58%	0.10%	
RBM10	0.00%	0.40%	
GNAS*	0.00%	0.00%	

*GNAS was included based on Level B evidence from CIViC (https://civicdb.org) indicating that the T393C genotype is associated with resistance to Gefitinib and Erlotinib.

Appendix Table S3.

NSCLC-related methylation markers

Methylation marker	Genomic coordinate	AUC in TCGA cohort
DMR1	chr1:91190560-91190680	0.987
DMR2	chr1:91196232-91196432	0.813
DMR3	chr12:85667516-85667696	0.950
DMR4	chr14:57265795-57266010	0.984
DMR5	chr2:63280989-63281463	0.956
DMR6	chr3:181421347-181421782	0.916
DMR7	chr8:72469370-72469490	0.924
DMR8	chr8:72469500-72469620	0.924

AUC was calculated to evaluate the performance of marker in differentiate tumor and normal tissue samples from TCGA cohort.

Appendix Table S4.

Tumor detection with standard DNA-seq and MM-seq

Cancer stage	Number of patients	Patients detected by standard DNA-seq	Patients detected by MM-seq
NSCLC	26	4 (15%)	7 (27%)
Early stage	18	0 (0%)	3 (17%)
Late stage	6	4 (67%)	4 (67%)
Stage unclear	2	0 (0%)	0 (0%)
Healthy control	13	0 (0%)	0 (0%)

Appendix Table S5.

Comparison of time cost in mutation and methylation identification between techniques

Experimental step	MM-seq	EM-seq	DNA-seq
End repair and ligation	2 h	2 h	2 h
Protective strand copy	1 h	-	-
Oxidization by TET2	2 h	2 h	-
Deamination by APOBEC	4 h	4 h	-
Index-PCR	1 h	1 h	1 h
Hybrid capture	18 h	18 h	18 h
Post-PCR	1 h	1 h	1 h
Total	29 h	50 h	

The time required for MM-seq to simultaneously capture mutation and methylation information was calculated, as well as the time needed for EM-seq and DNA-seq to identify mutations and methylation separately.
